# Standardized Single-Session Cardiopulmonary Exercise Test for Measuring Peak Oxygen Uptake, Oxygen On/Off Kinetics, and Skeletal Muscle Oxygenation in ICU Survivors—ICU Combined Assessment of Cardiorespiratory Exercise (ICU-CARE) Study: Protocol for a Cross-Sectional Observational Study

**DOI:** 10.2196/92346

**Published:** 2026-07-14

**Authors:** Britney Blunderfield, Jefferson Santana, Raju Majumdar, Isuru Herath, Marcus Blouw, Clare Ramsey, Kendiss Olafson, Mayson Sousa, J Gordon Boyd, Dmitry Rozenberg, Kristine Cowley, Margaret S Herridge, Jane Batt, Mac Horsburgh, Todd Duhamel, David Christiansen, Rodrigo Villar, Asher A Mendelson

**Affiliations:** 1 Department of Physiology and Pathophysiology, Rady Faculty of Health Sciences, University of Manitoba, Winnipeg, MB, Canada; 2 Cardiorespiratory and Physiology of Exercise Research Laboratory, Faculty of Kinesiology and Recreation Management, University of Manitoba, Winnipeg, MB, Canada; 3 Faculty of Kinesiology and Recreation Management, University of Manitoba, Winnipeg, MB, Canada; 4 Section of Critical Care, Department of Medicine, Rady Faculty of Health Sciences, University of Manitoba, Room GF-234, 820 Sherbrook St, Winnipeg, MB, Canada, 1 204-787-8059; 5 Program of Biomedical Engineering, Price Faculty of Engineering, University of Manitoba, Winnipeg, MB, Canada; 6 Section of Respirology, Department of Medicine, Rady Faculty of Health Sciences, University of Manitoba, Winnipeg, MB, Canada; 7 Department of Respiratory Therapy, College of Rehabilitation Science, Rady Faculty of Health Sciences, University of Manitoba, Winnipeg, MB, Canada; 8 Department of Critical Care Medicine, Queen's University, Kingston, ON, Canada; 9 Division of Respirology, Department of Medicine, Temerty Faculty of Medicine, University of Toronto, Toronto, ON, Canada; 10 University Health Network, Toronto, ON, Canada; 11 Interdepartmental Division of Critical Care Medicine, University of Toronto, Toronto, ON, Canada; 12 Patient Partner and ICU Survivor, Winnipeg, MB, Canada; 13 Institute of Cardiovascular Sciences, St. Boniface Hospital Albrechtsen Research Centre, Winnipeg, MB, Canada

**Keywords:** cardiopulmonary exercise test, constant work rate, on/off kinetics, near-infrared spectroscopy, peak oxygen consumption, ICU-acquired weakness, critical illness, exercise physiology

## Abstract

**Background:**

Intensive care unit–acquired weakness (ICU-AW) research focuses predominantly on intrinsic muscle pathology rather than integrated systemic interactions, which are commonly studied in exercise science. Peak oxygen uptake ($\dot{V}O_{2peak}$), $\dot{V}O_{2}$ on/off kinetics, and skeletal muscle oxygenation provide a quantitative evaluation of exercise capacity and are infrequently measured in intensive care unit (ICU) survivors. Routine cardiopulmonary exercise test (CPET) research separates $\dot{V}O_{2peak}$ and $\dot{V}O_{2}$ kinetics assessments into multiple sessions. Yet, a combined experimental approach may enhance diagnosis, follow-up retention, and mechanistic insight for patients with ICU-AW.

**Objective:**

This prospective cross-sectional observational study aims to develop a standardized, single-session CPET protocol for combined assessment of $\dot{V}O_{2peak}$, $\dot{V}O_{2}$ kinetics, and skeletal muscle microvascular oxygenation in ICU survivors, enabling quantitative and integrated assessment of ICU-AW.

**Methods:**

Adults mechanically ventilated for ≥7 days will be recruited to participate 6 months post-ICU discharge in a modified CPET exercise session on an upright cycle ergometer. The proposed standardization will involve (1) the estimation of $\dot{V}O_{2peak}$ using a priori formulas with a $\dot{V}O_{2peak}$ correction factor for the ICU population, (2) $\dot{V}O_{2}$ on-kinetics (constant work rate) targeting relative 30% $\dot{V}O_{2reserve}$, (3) an incremental ramp exercise based on self-reported functional status, and (4) a 10-minute recovery to quantify $\dot{V}O_{2}$ off-kinetics. In addition, near-infrared spectroscopy will be placed on the vastus lateralis muscle to simultaneously collect tissue saturation index and deoxyhemoglobin during each phase of the protocol.

**Results:**

Recruitment is anticipated to begin in June 2026 and is expected to be completed in 2029/2030. The anticipated sample size will be approximately 48 participants based on the feasibility of recruiting 1 participant per month and a sample size calculation based on Bland-Altman limits of agreement between predicted and measured $\dot{V}O_{2peak}$.

**Conclusions:**

The intensive care unit combined assessment of cardiorespiratory exercise (ICU-CARE) CPET protocol will enable the quantitative evaluation of $\dot{V}O_{2peak}$, $\dot{V}O_{2}$ on/off kinetics, and local microvascular skeletal muscle oxygenation during a single exercise session, facilitating the integrated physiological study of ICU-AW.

## Introduction

Intensive care unit–acquired weakness (ICU-AW)—defined as skeletal muscle and motor nerve dysfunction during and after critical illness [1]—remains a substantial contributor to long-term morbidity and loss of independence after prolonged intensive care unit (ICU) admission [2,3], occurring in 25% to 75% of mechanically ventilated patients [1,4]. Despite a high prevalence, there are currently limited tools to accurately quantify this disease burden and limited interventions to rehabilitate skeletal muscle after ICU discharge. The current approach for studying ICU-AW focuses on the pathological changes within the muscle [5], with minimal consideration for its in vivo physiological environment [6]. However, isolated skeletal muscle research may overlook the complex and integrated physiological systems contributing to ICU-AW. In addition, there is a paucity of research to describe the link between cardiovascular/respiratory dysfunction and ICU-AW, including how impairments lead to reduced exercise capacity and poor health outcomes.

The cardiopulmonary exercise test (CPET) is used clinically and in research to assess aerobic fitness and exercise capacity, predict health outcomes, diagnose cardiopulmonary dysfunction and functional limitations, and evaluate response to interventions [7]. The most common exercise metric for CPET is peak oxygen uptake $(\dot{V}O_{2peak}$), defined as the highest $\dot{V}O_{2}$ achieved during a test [8], particularly until the limit of tolerance (or willingness), signifying exercise capacity within this protocol. For many healthy and diseased populations, $\dot{V}O_{2peak}$ is a robust and comprehensive evaluation of global cardiovascular fitness and a strong predictor of mortality [9-11]. Additionally, $\dot{V}O_{2}$ kinetics assess the integrated capacity of the O_2_ transport pathway [12]. $\dot{V}O_{2}$ on-kinetics refers to the exponential rise of $\dot{V}O_{2}$ toward a steady state from rest to exercise below the lactate threshold, where the slope of the exponential rise represents the rate at which oxidative phosphorylation adjusts to a sudden increase in energy demand at a constant workload, reflecting both systemic oxygen transport and muscle metabolism [13]. $\dot{V}O_{2}$ off-kinetics refers to the exponential decline of $\dot{V}O_{2}$ postexercise [12], representing the rate of metabolic recovery, where oxygen uptake returns to baseline levels. Moreover, faster $\dot{V}O_{2}$ kinetics constitutes a small O_2_ deficit, less substrate-level phosphorylation, and greater exercise tolerance, whereas slower $\dot{V}O_{2}$ kinetics is linked to a higher O_2_ deficit, presenting a greater challenge to homeostasis, and is associated with poor exercise tolerance [12]. Quantification of these 3 variables—$\dot{V}O_{2}$_peak_, $\dot{V}O_{2}$ on- and off-kinetics—is critical to understanding the interplay between cardiopulmonary and muscular physiological mechanisms [13,14]. Together, they contribute to the overall phenotype of aerobic fitness, exercise efficiency, and exertional tolerance that impact quality of life and overall survival [13,15-17].

Research suggests that pathological conditions that affect cardiopulmonary function and muscle metabolic capacity are expected to alter $\dot{V}O_{2}$ kinetics [13]. Impediments at any upstream component along the oxygen delivery pathway (eg, lungs, cardiac, vascular, and microvascular) can attenuate $\dot{V}O_{2}$ kinetics, impairing exercise performance and impacting exercise tolerance [7,12,18,19]. While commonly used in clinical and research settings for evaluation of chronic cardiorespiratory diseases such as congestive heart failure, chronic obstructive pulmonary disease, or pretransplantation [20], CPET has not been widely adopted for assessment of ICU survivors after discharge. Past CPET studies in ICU survivors report reduced exercise capacity and limited aerobic fitness [5,7,21-24], yet underlying mechanisms for these findings, particularly beyond skeletal muscle biology, remain underexplored. Moreover, the evaluation of skeletal muscle microvascular oxygenation using near-infrared spectroscopy (NIRS) [25,26] has been notably lacking as a critical gap in our mechanistic understanding of ICU-AW. Overall, the paucity of quantitative and systematic data evaluating exercise capacity in ICU survivors is a key barrier to both understanding the pathophysiology underlying ICU-AW and to tailoring effective patient-centered rehabilitation strategies moving forward.

Typically, evaluation of $\dot{V}O_{2peak}$ and $\dot{V}O_{2}$ on/off kinetics requires multiple exercise sessions in order to first determine participant-specific $\dot{V}O_{2peak}$ and then to use these values to derive a constant work rate (CWR) as a percentage of $\dot{V}O_{2peak}$ or ventilatory thresholds [27-30]. However, this approach can limit recruitment and compliance, and is especially problematic for clinical research with vulnerable or disadvantaged participant groups [31]. Moreover, caregivers of ICU survivors are known to experience depressive symptoms for at least 1 year following discharge [32], which may also impact participation due to the inability or unwillingness to arrange and/or accompany to multiple sessions. Crucially, designing an efficient and simple protocol is critical for both the survivor and caregiver to foster recruitment and compliance. When $\dot{V}O_{2peak}$ and $\dot{V}O_{2}$ on/off kinetics are evaluated in a single session, as has been done by previous researchers [7,14,33], a uniform CWR is often prescribed regardless of fitness level and many times based on subjective parameters, which prevents accurate data comparison between participants. Furthermore, the timing of the phases of the exercise (ie, CWR, ramp, and recovery) within a single session protocol requires standardization, considering relatively short CWR or the implementation of an active recovery, which may compromise data quality for evaluating on/off kinetics [7,28].

Our team has recently developed a single-session CPET protocol for a combined assessment of $\dot{V}O_{2peak}$ and $\dot{V}O_{2}$ on/off kinetics in healthy young adults [28]. In this study, we showed that CWR intensity can be estimated a priori [16] from a Hansen-Wasserman population-based formulae of $\dot{V}O_{2peak}$ (mL kg^−1^ min^−1^), recommended by the American Thoracic Society (ATS) and the American College of Chest Physicians (ACCP) committee [17], relative to sex, body mass, measured $\dot{V}O_{2rest}$ (mL kg^−1^ min^−1^), multiplied by a desired intensity of 30 % $\dot{V}O_{2reserve}$. The estimated $\dot{V}O_{2peak}$ was in good agreement compared to real values measured later in the CPET, where participants achieved steady state (SS $\dot{V}O_{2}$) and adequate return to baseline during the CWR and recovery phases of the protocol.

We believe that this systematic methodological approach will be advantageous for studying exercise physiology in ICU survivors. The intensive care unit combined assessment of cardiorespiratory exercise (ICU-CARE) CPET protocol seeks to evaluate $\dot{V}O_{2peak}$, $\dot{V}O_{2}$ on/off-kinetics, and skeletal muscle oxygenation in ICU survivors. Our objective is to adapt our standardized, single-session CPET to ICU survivors, with the incorporation of skeletal muscle NIRS to better understand how systemic cardiovascular, respiratory, and skeletal muscle microvascular dysfunction contributes to ICU-AW. We hypothesize that this standardized single-session ICU-CARE CPET protocol is (1) feasible for recruitment and completion by ICU survivors and (2) suitable for standardized quantitative measurements of $\dot{V}O_{2peak}$, $\dot{V}O_{2}$ on/off kinetics, and skeletal muscle microvascular oxygenation. These results will enable comparison of exercise capacity, exercise tolerance, cardiorespiratory fitness, cardiopulmonary dysfunctions, and functional limitations between ICU survivors and healthy controls, and assess and track rehabilitation longitudinally after ICU discharge.

## Methods

### Ethical Considerations

This study was approved by the University of Manitoba Health and Institutional Research Ethics Boards (HS25592, SH2022-187, and RRC 2023/2119) and registered in ClinicalTrials.gov under the clinical study ID NCT06193980 (initial release 12/08/2023; last updated 06/11/2026). Written informed consent will be obtained on the day of the exercise testing by all participants.

### Study Design

The ICU-CARE CPET is a prospective cross-sectional observational study that will adhere to the STROBE (Strengthening the Reporting of Observational Studies in Epidemiology) guidelines [34] for final reporting of findings. This study follows the SPIRIT (Standard Protocol Items: Recommendations for Interventional Trials) statement [35], adapted for the purposes of an observational study.

The primary outcome for the study will be the feasibility of recruitment and completion of the protocol. Secondary outcomes of the study will be validation of the a priori equations used in the exercise protocol to calculate $\dot{V}O_{2peak}$ for the ICU population, and physiological observations derived from the study.

### Patient and Public Engagement

We have engaged with an ICU survivor, Mr Mac Horsburgh, as a member of our research team, who participated in research design to ensure our project is inclusive and aligns with lived experiences of critical illness. Specifically, we sought consultation on the importance of fitness as a long-term outcome that was given high priority for recovery after critical illness. Furthermore, Mr Horsburgh provided guidance on the accommodations that would be necessary for accessible research within the first 12 months following ICU discharge. We have also reviewed the protocol with Mr Horsburgh to ascertain his perceived capacity to undertake this study during the 12 months after his own ICU discharge. Moving forward, as participants are enrolled in the study, we plan to liaise with them and our identified patient partner in order to refine our experimental protocol and workflow.

### Study Settings

**Figure 1.**
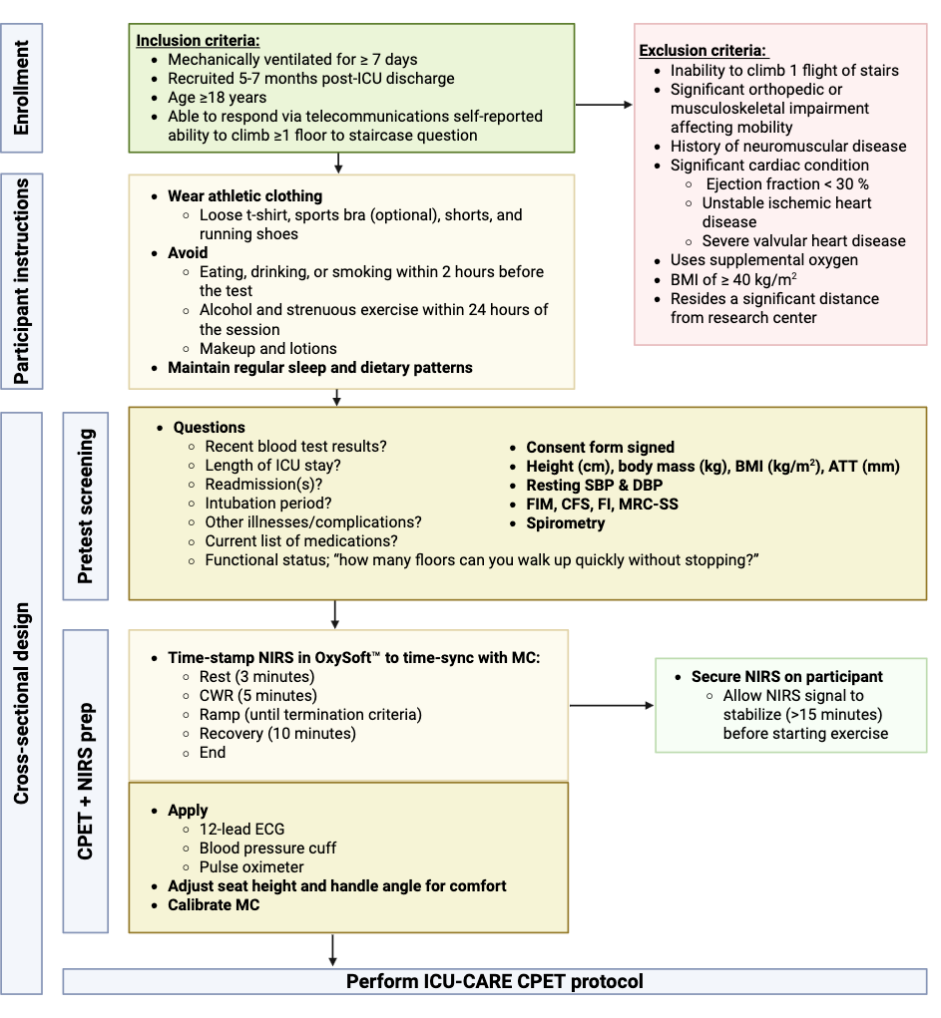
Flowchart for ICU-CARE CPET evaluation. ATT: adipose tissue thickness; CFS: Clinical Frailty Scale; CPET: cardiopulmonary exercise test; CWR: constant work rate; DBP: diastolic blood pressure; ECG: electrocardiogram; FI: Frailty Index; FIM: Functional Independence Measure; ICU: intensive care unit; ICU-CARE: intensive care unit combined assessment of cardiorespiratory exercise; MC: metabolic cart; MRC-SS: Medical Research Council sum score; NIRS: near-infrared spectroscopy; SBP: systolic blood pressure. Created in BioRender.

Data will be collected during a single visit to the Pulmonary Function Lab at Winnipeg Health Sciences Centre or St. Boniface Hospital in Winnipeg, Manitoba, Canada. Both sites have equivalent equipment for testing; the option for adding study sites is possible in the future, provided equipment and protocol harmonization. The flowchart for recruitment and evaluation prior to ICU-CARE CPET can be found in Figure 1.

### Inclusion and Exclusion Criteria

Participants will be patients of both sexes (18+ years) who have been mechanically ventilated for ≥7 days, consistent with the prior threshold for the development of ICU-AW [36], who voluntarily agree to participate, and have answered the self-reported functional ability questionnaire. Exclusion criteria will be defined as follows: those who self-report that they cannot climb at least one flight of stairs due to limited exercise capacity, have significant orthopedic or musculoskeletal impairment affecting mobility, have a medical history of neuromuscular disease, have ongoing respiratory limitations (ie, supplemental oxygen), have significant heart disease (ie, ejection fraction less than 30%, unstable ischemic heart disease, and severe valvular heart disease), have a BMI of ≥40 kg/m^2^ (impacting NIRS signal due to adipose tissue thickness) [37], or if the participant’s primary residence is a significant distance from the research center.

### Recruitment Strategy

Before ICU discharge, researchers will screen potential participants for willingness to join follow-up, meeting the inclusion criteria. After 3 months post-ICU discharge, participants will be contacted through telecommunication to determine their ongoing willingness and ability to participate in the ICU-CARE CPET study, with transportation compensation provided within the city limits. Participants will receive detailed information regarding procedures and potential risks before agreeing to attend (please refer to the Pre-CPET Evaluation section below). Due to the high degree of functional impairment and inherently dynamic transitions in care after ICU discharge, we intend for initial participation in the ICU-CARE CPET study to occur at 6 months after ICU discharge, with a range of ±1 month. Participants’ electronic medical records and telephone conversations will be screened for relevant hospital and postdischarge information regarding length of ICU stay, readmission(s), intubation period, concurrent illness, list of medications, and to acquire a recent blood test if available. Participants will then be asked, “How many floors can you walk up quickly without stopping?,” to prescribe the Watt/min increment for the ramp portion of the test (please see Incremental Phase below for more details). Participants will be given an option to be tested again at 12 months after ICU discharge to track their recovery, but this is not required for the main purpose of the study design.

### Pretest Instructions

To ensure data accuracy, participants should adhere to specific pretest instructions through telecommunications leading up to the session, including wearing athletic clothing such as a loose-fitting T-shirt, shorts, and running shoes; avoiding eating, drinking, or smoking within 2 hours before the test; avoiding alcohol consumption and strenuous exercise 24 hours prior to the appointment; avoiding makeup and lotions; and maintaining regular sleep and dietary patterns the day before the test.

### Pre-CPET Evaluation

All potential risks of the procedure will be restated prior to starting the session, and written informed consent will be obtained on the day of the CPET test. According to the ATS, exercise testing is very safe with minimal risk (<0.1%) of cardiac event, and extremely low risk (<0.01%) of death [17]. CPET is routinely undertaken for patients with advanced cardiorespiratory disease, those being evaluated for heart [38] and lung transplantation [39], in advanced heart failure management [40], and in ICU follow-up within the same time period as proposed in this study [5,7,24,41].

Resting systolic (SBP_rest_) and diastolic (DBP_rest_) blood pressure and heart rate (HR_rest_) will be measured prior to exercise, following ATS/ACCP guidelines [17]. ICU-AW will be assessed using the manual testing of muscle strength and severity using the Medical Research Council sum score (MRC-SS) [42], a tool commonly used to diagnose and track the development of ICU-AW [43,44], and the Functional Independence Measure (FIM) questionnaire to assess their ability to carry out activities of daily living [36,45]. FIM score—even early after ICU discharge—has been shown to predict long-term disability in a cohort of ICU survivors [36]. The researchers will also administer the Clinical Frailty Scale, a simple clinician-based tool to quickly evaluate the overall frailty score based on a 9-point scale, and the Frailty Index, which offers a detailed, multidimensional assessment of specific deficits in health and function—both previously validated in the critically ill population [46,47]. Spirometry will be performed prior to beginning the CPET exercise protocol.

### Equipment

The ICU-CARE CPET protocol will be performed using an upright cycle ergometer (Lode Corival | CPET, Lode BV Medical Technology). The pulmonary oxygen uptake ($\dot{V}O_{2}$), carbon dioxide production ($\dot{V}{CO}_{2}$), and the respiratory exchange ratio of $\dot{V}{CO}_{2}$/$\dot{V}O_{2}$ will be collected, monitored, and reported mid-5 of 7 breaths averaged using the Ultima CardiO2 gas exchange analysis system metabolic cart (MGC Diagnostics). A fitted air-cushioned face mask (preVent) and a bidirectional breath-by-breath volume sensor (preVent) will be used to collect the ventilatory variables. The Ultima CardiO2 will be calibrated before each test by using automated gas and flow calibrations as well as quality assurance to ensure compliance with the procedures recommended by the ATS/ERS pulmonary function acceptability and repeatability guidelines, as described in the user manual. Heart rate will be recorded using a 12-lead Mortara electrocardiogram (ECG) embedded in the Ultima CardiO2 system [48]. Blood pressure will be recorded every 2 minutes using the Tango M2 Stress Test Monitor (SunTech Medical) system during the exercise test.

NIRS is a noninvasive optical technology that provides information on microvascular oxygen delivery and local tissue utilization in skeletal muscle during exercise [49,50]. This technology provides insight into the concentration and oxygenation of biological tissues through the status of light-absorbing chromophores, such as hemoglobin (Hb) and myoglobin (Mb) within the near-infrared region (700‐900 nm) penetrating several millimeters [25]. The NIRS device (PortaMon, Artinis Inc) will be placed on the vastus lateralis muscle [51-53], about 12‐14 cm above the knee, secured by a band to minimize motion artifacts during cycling exercise. Adipose tissue thickness will be measured over the vastus lateralis site using Sonosite PX Ultrasound System (FUJIFILM Sonosite, Inc).

### Safety and Termination Criteria

Medical staff and researchers will be on-site for continuous monitoring to ensure participants’ safety throughout the exercise protocol. ICU-CARE CPET will be terminated if participants meet the formal criteria as outlined in the standard ATS/ACCP guidelines [17], which include chest pain suggestive of ischemia, ischemic ECG changes, complex ectopy, second- or third-degree heart block, a fall in systolic pressure by 20 mm Hg from the highest value during the test, hypertension (250 mm Hg systolic, 120 mm Hg diastolic), severe desaturation (indicated by pulse oximetry, arterial oxygen saturation [Sp_O2_] 80% with symptoms of severe hypoxemia), sudden pallor, loss of coordination, mental confusion, dizziness or presyncope, and signs of respiratory failure [17]. The test will also be terminated immediately if participants request to stop (volitional exhaustion) or if they exhibit any of the following: significant discomfort preventing continued exercise, abnormal pain, shortness of breath limiting exercise continuation, light-headedness, dizziness, or inability to maintain 60 rpm (revolutions per minute) cadence (±10 rpm).

### Data Monitoring Committee

The research staff will report details and deviations of the data collection process and subsequent analyses to the local research ethics board. Because this is a small observational study, we are not planning an interim analysis, nor will there be a formal data monitoring committee outside of the research team.

### ICU-CARE CPET Protocol

**Figure 2.**
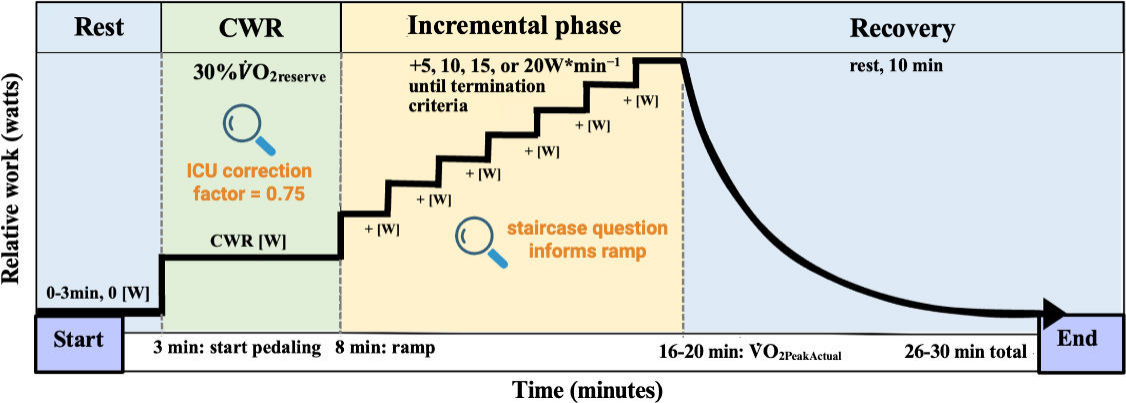
Illustration of the ICU-CARE CPET protocol for ICU survivors using an upright cycle ergometer. Orange words represent the protocol adaptations from Santana et al [28] study in healthy controls. CPET: cardiopulmonary exercise test; CWR: constant work rate; ICU: intensive care unit; ICU-CARE: intensive care unit combined assessment of cardiorespiratory exercise; V̇O_2peak_: peak oxygen uptake; V̇O_2reserve_: oxygen uptake reserve. Created in BioRender.

The ICU-CARE CPET protocol (Figure 2) will combine measurements of $\dot{V}O_{2peak}\dot{,V}O_{2}$ on/off kinetics, as previously described by our research team in healthy participants [28], with the addition of two adaptations for ICU survivors. Before the start of this protocol, participants’ seat height and handle angle will be adjusted for comfort. The protocol will be composed of a baseline phase, a CWR phase (on-kinetics), an incremental phase (peak), and a recovery phase (off-kinetics), as described below.

#### Rest Phase

The rest phase consists of 3 minutes baseline, seated on the upright cycle ergometer without pedaling to establish baseline values for $\dot{V}O_{2}$ and NIRS signals prior to initiating the CWR phase.

#### Constant Work Rate Phase (On-Kinetics)

The CWR phase will entail a 5-minute steady-state moderate-intensity upright cycling exercise at the desired intensity of 30%, relative to the individual’s estimated $\dot{V}O_{2reserve}$ (as described below in the .$\overset{˙}{\;V}O_{2reserve}$Calculation section) to perform exercise, with a cadence of 60 rpm [28]. In a recent publication, our team used a 4-step process to standardize and determine the optimal CWR exercise intensity during a CPET protocol [28]. We will adopt and adapt these steps to derive a CWR intensity for ICU survivors, as outlined below.

##### ICU Survivor Prediction Equation for$\dot{V}O_{2peak}$

In this first step, to determine CWR, a recent Canadian adult (≥40 years of age) normative (50th percentile) and sex-specific equation (Equation 1) [54] will be used to predict $\dot{V}O_{2peak}$. Subsequently, an ICU correction factor of 0.75 (ICU0.75) is to be multiplied based on an average of 25% $\dot{V}O_{2peak}$ reduction in ICU survivors, previously described in the literature [5,7,21].


(1) $$\begin{array}{rl} \text{Estimated }\dot{V}O_{2\text{peak}} & =(\left(0.012905699\cdot H)+(0.007291081\cdot BM\right)\\ \;+(0.564188602\cdot S)-(0.016521850\cdot A)\\ \;-0.076072067)\times ICU0.75 \end{array}$$

where age in years, BM = body mass (kg), H = height (cm), S = sex: male = 1, female = 0, and ICU0.75 = ICU correction factor of 0.75.

##### $\dot{V}O_{2reserve}$ Calculation

$\dot{V}O_{2reserve}$ will be calculated as the difference between the estimated $\dot{V}O_{2peak}$ from Equation 1 and a fixed $\dot{V}O_{2rest}$ of 3.5 mL kg^−1^ min^−1^ (to promote efficiency within a clinical setting) to calculate $\dot{V}O_{2reserve}$ before participant arrival.

$\overset{˙}{V}O_{2reserve}=\overset{˙}{V}O_{2peak}-\overset{˙}{V}O_{2rest}$

##### $\dot{V}O_{2CWR}$ Calculation

The target $\dot{V}O_{2CWR}$ will be determined as described in Equation 2, in which the $\dot{V}O_{2reserve}$ obtained in step 2, described above, will be multiplied by the desired intensity (30%).


(2) $$\begin{array}{rl} \dot{V}O_{2CWR} & =\left[\left(\dot{V}O_{2\text{peak}}-\dot{V}O_{2\text{rest}})\times (\%\text{desired intensity}\right)\right]+\dot{V}O_{2\text{rest}} \end{array}$$

where $\dot{V}O_{2peak}$ (mL kg^−1^ min^−1^) is the value estimated in step 1 for ICU survivors; $\dot{V}O_{2rest}$ (mL kg^−1^ min^−1^) is the value of oxygen uptake at rest; and % desired intensity, expressed in decimals, is the desired intensity (relative to the individual’s $\dot{V}O_{2reserve}$) to perform the exercise. The 30% $\dot{V}O_{2reserve}$, a light intensity according to the American College of Sports Medicine [55], optimizes conditions so that participants perform the CWR exercise phase below the ventilatory threshold, to achieve a steady-state $\dot{V}O_{2}$ within 5 minutes, while minimizing interference with the subsequent progressive incremental phase of the protocol.

##### Estimation of Work Rate in Watts

The work rate (watts) will be obtained by using the individual’s body mass and the $\dot{V}O_{2}$ (mL kg^−1^ min^−1^), as described in Equation 3 [55].


(3) $$\begin{array}{rl} \text{Work rate [W]} & =\frac{\text{Body mass}\times \left(\dot{V}O_{2\text{CWR}}-7\right)}{1.8}\times 0.164 \end{array}$$

where the individual’s body mass will be expressed in kilograms (kg) and the $\dot{V}O_{2}$ (mL kg^−1^ min^−1^) is the target $\dot{V}O_{2CWR}$ obtained in step 3 (Equation 2). The result of Equation 3 will determine the CWR (Watts) performed during the ICU-CARE CPET protocol on the cycle ergometer.

### Incremental Phase

The incremental phase of the protocol will be based on the information obtained from the self-reported staircase question [56] and modified Medical Research Council (mMRC) dyspnea scale [57] during the recruitment process. For the staircase question, participants will be asked, “How many floors can you walk up quickly without stopping?,” during the recruitment process. Based on their responses, the increments will be defined as follows: one floor (5 W min^−1^; 50 W), two floors (10 W min^−1^; 100 W), three floors (15 W min^−1^; 125‐150 W), and four floors (20 W min^−1^; 200 W). For the mMRC dyspnea scale, participants’ severity of breathlessness will be reported based on activity descriptors and mMRC grade between 0 and 4 with their respective Watt equivalent, as described in Table 1. Participants will cycle until the termination criteria are met, as described above.

**Table 1.** Modified Medical Research Council (mMRC) dyspnea scale.

Grade	Activity descriptor	Ramp
0	Strenuous activity, not troubled by breathlessness (eg, cycling and shoveling heavy snow)	20 W min^−1^
1	Walking up an incline causes shortness of breath	15 W min^−1^
2	Walking two blocks and experiencing breathlessness	10 W min^−1^
3	Walking short distances and needs to catch breath (eg, 100 ft, or room to room)	10 W min^−1^ for males, 5 W min^−1^ for females
4	Cannot get dressed or shower without shortness of breath	No CPET^a^

a CPET: cardiopulmonary exercise test.

### Recovery Phase (Off-Kinetics)

After the cessation of exercise, participants will stop pedaling, remain seated on the cycle ergometer, and start passive recovery for 10 minutes. $\dot{V}O_{2}$, deoxyhemoglobin (HHb), and tissue saturation index (TSI) responses will be used to evaluate off-kinetics.

Figure 2 displays the ICU-CARE CPET protocol including (1) resting phase: 3 minutes baseline $\dot{V}O_{2}$ measurements, no pedaling, seated on the bike; (2) constant work rate phase (CWR): 5 minutes pedaling at 30%$\overset{˙}{\;V}O_{2reserve}\;\left(\overset{˙}{V}O_{2peak\;predicted}-\overset{˙}{V}O_{2rest}\right)$, whereby $\dot{V}O_{2peakpredicted}$ is estimated a priori using population-based estimates and an ICU $\dot{V}O_{2peak}$ multiplied by a correction factor of 0.75; (3) incremental phase: $\dot{V}O_{2peak}$ is assessed through a ramped cycling exercise, based on self-reported functional status (5, 10, 15, or 20 W min^−1^), until termination criteria are met (as described in the Safety and Termination Criteria section above); and (4) recovery: 10-minute, no pedaling.

### Data Analysis

Similar to the $\dot{V}O_{2}$ analysis conducted on healthy adult populations [28], $\dot{V}O_{2}$ on-kinetics (rest-to-exercise transition), $\dot{V}O_{2peak}$, and $\dot{V}O_{2}$ off-kinetics (exercise-to-recovery transition) will be analyzed in the ICU-CARE CPET within a single bout—reporting the measured and predicted $\dot{V}O_{2peak}$ (mL kg^−1^ min^−1^), measured SS $\dot{V}O_{2}$ at CWR (mL kg^−1^ min^−1^), “actual” calculated 30% $\dot{V}O_{2reserve}$ (mL kg^−1^ min^−1^), and targeted intensity achieved at CWR as a percentage (%). Additionally, NIRS will be analyzed to evaluate skeletal muscle microvascular oxygenation. The primary variables will be pulmonary $\dot{V}O_{2}$, deoxyhemoglobin (HHb; deoxy[Hb+ Mb]), and TSI ($\frac{\left[oxy\left(Hb+Mb\right)\right]}{\left[oxy\left(Hb+Mb\right)\right]+\left[deoxy\left(Hb+Mb\right)\right]}*100$), measured noninvasively at a sample rate of 10 Hz.

Skeletal muscle oxygenation measured by NIRS (Portamon, Artinis Medical Systems) will be transmitted wirelessly into OxySoft software, time-synched with the metabolic cart with real-time impulse by inserting key commands on the NIRS computer (listed in Figure 1). On OxySoft, export NIRS data to Microsoft Excel, then adjust to timestamps as described above and illustrated in Figure 2. For a combined analysis, $\dot{V}O_{2}$ and NIRS data will be preprocessed using a customized LabVIEW code (LabView v20.0.01, National Instruments Corp), specifically designed to perform the required analyses. The first analysis removes noise and motion artifacts. Initially, outliers will be removed by applying 2 standard deviations in a window size of 7 seconds, then applying a 350-second moving average, linearly interpolated to provide second-by-second values [52]. Then, the data is resampled at 1 Hz and time-aligned with each transition to be synchronized with the metabolic cart.

With exercise starting at zero, subsequent analysis will be performed using a mono-exponential model to fit the pulmonary or primary component of the $\dot{V}O_{2}$ response (ie, phase II) by skipping the cardiogenic phase (ie, phase I), as previously recommended [33,58], left-shifting at 20 s in length [52]. Then, the steady-state $\dot{V}O_{2}$ (SS $\dot{V}O_{2}$ or phase III) will be calculated as the average of the last 2 minutes of the CWR exercise phase [28]. The mono-exponential fitting is characterized by $\dot{V}O_{2}$ parameters, including tau (τ), time delay (TD), amplitude A_0_ and A_1_, and mean response time (MRT), where τ is defined as the time constant of the exponential function, representing the time taken to reach 63% of the steady-state or final value; TD records the delay to the exponential response start (ie, phase II) (the model is not constrained to pass through the origin) before oxygen uptake responds to exercise; A_0_ represents the baseline value (resting $\dot{V}O_{2}$), whereas A_1_ represents the change of amplitude from baseline to steady-state $\dot{V}O_{2}$ (ie, SS $\dot{V}O_{2}$ – baseline value); and MRT, defined as TD + τ, representing the overall time to adjust the rate of $\dot{V}O_{2}$ to changes in workload [28]. At the end of the incremental phase, $\dot{V}O_{2peak}$ will be defined as the highest value of the last 30 seconds before volitional exhaustion after applying a 20-second moving average [59,60]. The quality of the fitting will follow our previous protocol [28]. The analysis of residuals, the degree of linear correlation between the experimental data and fitted function (*r*), the 95% CI band, and the significance level (*P* value) of the estimated parameters are used to determine the quality of the fitting.

The HHb signal will be normalized using a 60-second average before the exercise begins, then analyzed by detecting the nadir, occurring after a sudden decrease in HHb signal, to exclude data not associated with muscular $\dot{V}O_{2}$ dynamics, referring to the muscle pump [61]. After the nadir, HHb increases monoexponentially, representing the $\dot{V}O_{2}$ dynamics during the onset of exercise [27,62-64].

The HHb monoexponential function is expressed in the equation below:


$$HHb\;{\left(t\right)}^{\;}=HHb_{bl}+a\left[1-e^{-\left(t-TD\right)/\tau }\right]$$

where HHb_bl_=HHb baseline, *a*=amplitude, *e*=mathematical constant, *t*=time, TD=time delay, and τ=time constant.

MRT of the HHb is also estimated by the sum of τ and TD, to determine the MRT time constant.

Changes in TSI will be assessed by calculating the difference between baseline (average 30 seconds before CWR) and end of exercise (30-second average before start of recovery) to quantitatively evaluate tissue saturation fluctuation of oxygen uptake within the skeletal muscle, reported as ΔTSI [%] in the equation below:


$$\Delta TSI\;\left[\%\right]={TSI}_{peak}-{TSI}_{bl}$$

where TSI_peak_=30-second average before $\dot{V}O_{2peak}$; TSI_bl_=TSI baseline, 30-second average before commencing exercise.

Secondary variables from the CPET will be collected in an exploratory manner. These include carbon dioxide output ($\dot{V}{CO}_{2})$, respiratory exchange ratio, oxyhemoglobin, heart rate response, peak oxygen pulse, tidal volume, breathing frequency, minute ventilation (VE), ventilatory efficiency (VE/VCO_2_), and breathing reserve. Measured adipose tissue thickness of ≥12.5 mm will define a subgroup of patients with less reliable skeletal muscle NIRS measurements [37]; sensitivity analysis will be undertaken with both inclusion and exclusion of this subgroup in cohort outcomes. Other variables to consider include the CPET time of completion, patient-reported outcomes, and any other additional comments regarding the ICU-CARE CPET protocol experience.

### Missing Data

Missing data, if any, will be reported with an explanation upon publication.

## Results

### Outcome Measures

Our primary methodological outcome for this study will be the feasibility of recruitment and completion of the protocol. The secondary outcome will be validation of a priori physiological equations through assessment of agreement between predicted and measured variables of $\dot{V}O_{2peak}$ and $\dot{V}O_{2CWR}$. Physiological observations of exercise capacity and skeletal muscle oxygenation derived from the protocol will also be considered secondary outcomes.

### Anticipated Feasibility of Participation and Study Protocol Completion

Recruitment is anticipated to begin in June 2026 and is expected to be completed in 2029/2030. Two patients have participated in a prior pilot version of the protocol in 2025, which involved testing the phases of the exercise protocol with commercial population-based equations and application of the NIRS during exercise. This was done to determine equipment functionality, data quality, transition between CPET phases, and sample values of $\dot{V}O_{2peak}$ in the ICU population; results from these pilot participants will not be used in the main study. Based on this pilot, we have incorporated a contemporary standardized population-based equation for estimation of $\dot{V}O_{2peak}$ (Equation 1). The protocol registry has been amended to align with the primary and secondary outcomes listed herein, and the removal of unrelated outcomes and analyses from prior study iterations.

We expect to screen 1‐2 patients per week in the ICU, based on our local ICU data of approximately 1350 admissions per year, 68% bed utilization of mechanical ventilation, and a median duration of mechanical ventilation of 5.8 days. Of the screened patients, we aim for 50% enrollment to participate in follow-up via telephone after ICU discharge. Based on prior studies evaluating ICU-AW [45], we expect a significant loss to follow-up and/or inability to return to the clinic for the study. We are targeting a feasibility of enrollment rate of 1 participant per month throughout the study duration to generate preliminary outcome measurements to inform future studies. Of the individuals who are participating, our protocol feasibility metric will be ≥80% completion of all phases of the ICU-CARE CPET, enabling integrated evaluation of $\dot{V}O_{2peak}$, $\dot{V}O_{2}$ on/off kinetics, and local microvascular oxygenation to provide quantitative evaluation of exercise capacity and health outcomes in the ICU survivor population.

### Validation of ICU-Specific Protocol Adaptations

We will determine whether our ICU correction factor of 0.75 is an acceptable method for estimating $\overset{˙}{V}O_{2peak}$ from population equations. The statistical analysis for comparisons between predicted versus measured variables—specifically $\dot{V}O_{2peak}$ and $\dot{V}O_{2CWR}$—will be performed with (1) percentage differences between predicted and measured variables for general weighted inspection of the difference, (2) linear regression/correlational analysis with measured variables as the dependent and predicted as the independent variable, (3) Bland-Altman analysis [65] to assess the agreement between predicted and measured variables, and (4) Cohen *d* test to assess the size of the difference/similarity between predicted and measured variables; the difference between measured and predicted values will be considered clinically significant (ie, discordant) if they exceed a Cohen *d* value of 0.5. The statistical significance level will be set at *P*<.05.

### Evaluation of Exercise Capacity in ICU Survivors

The ICU-CARE CPET protocol provides critical insights into both systemic cardiorespiratory dynamics that can influence exercise capacity [5,7,21-24,41] and local microvascular insights provided by NIRS technology [49,50] to quantitatively assess cardiopulmonary and skeletal muscle oxygenation responses during exercise. Data will be reported as mean (SD) or mean (95% CI) for the cohort, as well as disaggregated according to biological sex. The quality of the fitting will be assured by the analysis of residuals, the degree of linear correlation between the experimental data, fitted function (*r*), and the significance level (*P* value) of the estimated parameters. We expect that the standardized, single-session ICU-CARE CPET protocol proposed in this study will enable (1) quantitative evaluation of $\dot{V}O_{2peak}$, $\dot{V}O_{2}$ on/off kinetics, and skeletal muscle microvascular oxygenation in ICU survivors; (2) delineation of physiological relationships between the above variables to characterize the mechanisms of ICU-AW, including physiological heterogeneity between patients; (3) prospective longitudinal assessment of rehabilitation trajectory of individual ICU survivors after critical illness; and (4) comparison with other populations (eg, healthy, older adults, and chronic diseases).

### Statistical Analysis and Sample Size Calculation

In order to calculate a sample size for this study based on statistical testing, we have selected the Bland-Altman agreement [65] between predicted and measured $\dot{V}O_{2peak}$ as the target variable of interest. This was chosen because it is the first outcome variable that is being calculated based on a priori equations and the ICU correction factor (see Equation 1). We estimate sample size using the method described by Lu et al [66], whereby a sample size of 48 participants is needed to detect a standardized agreement limit (*δ*/*σ*) of 2.9 and a standardized difference limit (*μ*/*σ*) of 0.2, with *α*=0.05 and *β*=0.2 (80% power). With this method, *δ* is the maximum acceptable agreement limit, *μ* is the mean difference, and *σ* is the standard deviation of differences between predicted and measured $\dot{V}O_{2peak}$. Because this is the first application of these physiological equations, the true *σ* of the population will only be ascertained after study completion, and will inform sample size calculation for future studies. Further testing of secondary statistical outcomes, including sex-disaggregated and sensitivity analysis, is not directly informed by sample size and should be considered hypothesis generating. As a result, these subgroups will likely be underpowered until evaluation in larger studies.

For longitudinal testing of the same participant at different timepoints (ie, 6 and 12 months), changes in quantitative exercise, kinetic, and NIRS variables will be reported as absolute and %change between sessions; statistical differences between sessions will be calculated with the Wilcoxon signed-rank test, with a significance level set at *P*<.05. Because only a subgroup of participants will be completing both sessions, the results from this longitudinal analysis will be used to inform the design of future studies.

### Dissemination Policy

Research staff will communicate the results of exercise testing to participants at the time of their test. The researcher will then offer a digital copy of the CPET printed from the metabolic cart sent via email, excluding confidential information (eg, name). Results of the study are anticipated to be published when the study is completed.

## Discussion

### Rationale for Protocol Development

This study introduces the ICU-CARE CPET: a standardized protocol combining $\dot{V}O_{2peak}$, $\dot{V}O_{2}$ on/off kinetics, and skeletal muscle microvascular oxygenation measurements in ICU survivors. Designed to overcome limitations of traditional exercise methodology [28] (eg, multiple hospital/research lab visits, longer data collection, recruitment, and compliance issues), and recognizing that this field is evolving [7,28], we propose to combine CPET with $\dot{V}O_{2}$ kinetics, improving overall research efficiency within clinical settings [7], empowering further investigation of ICU-AW and its associated pathophysiology.

Expanding beyond a focus on skeletal muscle biology, ICU-AW is now believed to be multifactorial [1,22,67,68]. As demonstrated with other diseases such as congestive heart failure, we propose that the syndrome of ICU-AW (ie, impaired locomotive capacity) likely represents a failure of integrated systems that coordinate oxygen delivery, terminal microvascular-myocyte oxygen exchange, and skeletal muscle mitochondrial oxygen utilization [69]. This point of view has led to a shift of research from local skeletal muscle biology toward global investigation of the integrative physiologic responses across the oxygen delivery pathway [6]. Consequently, we are proposing the incorporation of CPET and NIRS to quantitatively assess cardiopulmonary and skeletal muscle microvascular oxygenation responses during exercise.

Currently, there is a lack of standardization for combining assessments of $\dot{V}O_{2peak}$, $\dot{V}O_{2}$ on/off kinetics, and skeletal muscle NIRS post-ICU discharge. This protocol was developed based on the study of Longobardi et al [7] and our own work with healthy control populations [28], both exploring single-session CPET for the combined assessment of $\dot{V}O_{2peak}$ and on/off kinetics. Longobardi et al [7] used a single-session treadmill CPET incorporating a 3-minute moderate-intensity walk (below ventilatory anaerobic threshold) before the incremental phase and a 6-minute recovery period in survivors of severe COVID-19 3‐6 months after hospitalization (n=53). The limitations of their study were the uniform application of the CWR power for all participants, the relatively short period of time for on/off kinetics, which may not achieve steady state, and the absence of NIRS measurements to evaluate skeletal muscle oxygenation. We expanded on this protocol with a standardized and single-session cycle ergometer protocol and tested it in healthy young adults (n=20). This CPET protocol consisted of 3 minutes of rest seated in a chair ($\dot{V}O_{2}rest)$, 3 minutes of baseline seated on the cycle ergometer (no pedaling), 5 minutes of CWR at 30% estimated $\dot{V}O_{2reserve}$ (60 rpm), incremental phase (20 W min^−1^) to attain $\dot{V}O_{2peak}$, and 10 minutes of passive recovery. Based on the results, it was concluded that the proposed protocol was feasible and appropriate for the evaluation of the intended variables. Although not included in our healthy control study, NIRS data were also collected and are being analyzed separately to ensure data quality and reliability.

Research suggests that 1 of 3 ICU survivors experience reduced $\dot{V}O_{2peak}$ [21], often falling below 80%‐85% of predicted values [5,21,22] during the first year after ICU discharge. Based on these studies, we have decided to use a correction factor of 0.75 (75%) to account for the reduced $\dot{V}O_{2peak}$ in the ICU survivor population. This is incorporated into Equation 1 (in step 1) and will be evaluated during this study. We have also adapted the incremental phase of this protocol (W) based on patients’ self-reported functional status (5, 10, 15, or 20 W min^−1^), derived from a practical guide for CPET [56], suggesting the use of the staircase question to inform ramp modifications. Research suggests a linear relationship shared between the number of steps climbed, minute ventilation, and $\dot{V}O_{2peak}$ [68]. Our practical staircase question [56] will be compared with the mMRC using a correlational analysis within the prospective study, offering a tailored approach to promote inclusion. In future studies, analysis of the statistical significance correlation between mMRC and staircase question steps can be conducted to assess its relationship, depending on sample size, to get a significant result, to validate the staircase question to be used instead of the mMRC scale, or in conjunction.

### Future Directions

This protocol allows for a comparative analysis between ICU survivors and other populations (eg, healthy controls), establishing a foundation for future investigation into the pathophysiology underlying ICU-AW and its relationship to long-term health outcomes. The prospective, observational, cross-sectional ICU-CARE study will be initially conducted in two ICU sites in one city (Winnipeg) to assess feasibility and to evaluate the accuracy and validity of the a priori physiological equations used in this study. Then, limitations will be addressed within future research studies across multiple sites in Canada. This protocol is designed to accommodate a wide range of ICU survivors; however, variability between participants (eg, comorbidities and exercise capacity before and after ICU) may impact the generalizability of results. Future research could also investigate cardiovascular and respiratory responses in greater depth, and expand to larger and more diverse cohorts. Furthermore, longitudinal testing will be required to evaluate intra- and interparticipant reproducibility over repeated sessions to ensure protocol robustness.

### Conclusions

The development of the ICU-CARE CPET protocol offers a novel, standardized, single-session approach to quantify both systemic and local physiological impairments associated with ICU-AW. In the modified CPET exercise session, the proposed protocol standardization involves (1) the estimation of $\dot{V}O_{2peak}$ using a priori formulae with a $\dot{V}O_{2peak}$ ICU correction factor, (2) on-kinetics (CWR) targeting relative 30% $\dot{V}O_{2reserve}$, (3) an incremental ramp exercise based on self-reported functional status, and (4) a 10-minute recovery to obtain off-kinetics. Within this combined CPET-NIRS framework, this protocol is anticipated to permit submaximal exercise and peak exercise evaluation during the same session, enabling the quantitative assessment of $\dot{V}O_{2peak}$, $\dot{V}O_{2}$ on/off kinetics, and local microvascular skeletal muscle oxygenation. Together, these metrics offer a valuable methodological framework for advancing ICU-AW research to enhance our understanding of its underlying mechanisms, while also enabling new avenues in ICU follow-up research to further improve treatment and rehabilitation strategies.

## Abbreviations

ACCP: American College of Chest Physicians
ATS: American Thoracic Society
CPET: cardiopulmonary exercise test
CWR: constant work rate
ECG: electrocardiogram
ERS: European Respiratory Society
FIM: Functional Independence Measure
Hb: hemoglobin
HHb: deoxyhemoglobin
ICU: intensive care unit
ICU-AW: intensive care unit–acquired weakness
ICU-CARE: intensive care unit combined assessment of cardiorespiratory exercise
Mb: myoglobin
MRC-SS: Medical Research Council sum score
MRT: mean response time
NIRS: near-infrared spectroscopy
rpm: revolutions per minute
SPIRIT: Standard Protocol Items: Recommendations for Interventional Trials
V̇O_2_: steady-state oxygen uptake
STROBE: Strengthening the Reporting of Observational Studies in Epidemiology
TD: time delay
TSI: tissue saturation index
V̇CO_2_: carbon dioxide production
V̇O_2_: oxygen uptake
V̇O_2peak_: peak oxygen uptake
V̇O_2reserve_: oxygen uptake reserve
